# Ontogenetic changes in the body plan of the sauropodomorph dinosaur *Mussaurus patagonicus* reveal shifts of locomotor stance during growth

**DOI:** 10.1038/s41598-019-44037-1

**Published:** 2019-05-20

**Authors:** Alejandro Otero, Andrew R. Cuff, Vivian Allen, Lauren Sumner-Rooney, Diego Pol, John R. Hutchinson

**Affiliations:** 1 División Paleontología de Vertebrados, Museo de La Plata, Paseo del Bosque s/n, (1900) La Plata, Argentina; 2 CONICET - Consejo Nacional de Investigaciones Científicas y Técnicas, Ciudad Autónoma de Buenos Aires, Argentina; 3 Structure and Motion Laboratory, Department of Comparative Biomedical Sciences, The Royal Veterinary College, Hatfield, Hertfordshire, United Kingdom; 4 Oxford University Museum of Natural History, Oxford, United Kingdom; 5 Museo Paleontológico “Egidio Feruglio”, Trelew, Argentina

**Keywords:** Software, Palaeontology

## Abstract

Ontogenetic information is crucial to understand life histories and represents a true challenge in dinosaurs due to the scarcity of growth series available. *Mussaurus patagonicus* was a sauropodomorph dinosaur close to the origin of Sauropoda known from hatchling, juvenile and mature specimens, providing a sufficiently complete ontogenetic series to reconstruct general patterns of ontogeny. Here, in order to quantify how body shape and its relationship with locomotor stance (quadruped/biped) changed in ontogeny, hatchling, juvenile (~1 year old) and adult (8+ years old) individuals were studied using digital models. Our results show that *Mussaurus* rapidly grew from about 60 g at hatching to ~7 kg at one year old, reaching >1000 kg at adulthood. During this time, the body’s centre of mass moved from a position in the mid-thorax to a more caudal position nearer to the pelvis. We infer that these changes of body shape and centre of mass reflect a shift from quadrupedalism to bipedalism occurred early in ontogeny in *Mussaurus*. Our study indicates that relative development of the tail and neck was more influential in determining the locomotor stance in Sauropodomorpha during ontogeny, challenging previous studies, which have emphasized the influence of hindlimb vs. forelimb lengths on sauropodomorph stance.

## Introduction

One of the most dramatic evolutionary transformations recorded in the history of terrestrial vertebrates is the acquisition of gigantic body size in sauropods, through the transition from small to mid-sized early sauropodomorphs (dinosaurs that were mostly bipedal, omnivorous, and weighed less than 3 tons) to giant eusauropods (large, graviportal and quadrupedal herbivores of more than 10 tons). Key aspects of the origin of Eusauropoda included transformations of the postcranial skeleton that supported their gigantism, which is correlated with heavily built (graviportal) limbs indicating obligatory quadrupedal stance and columnar limb posture^1–4^. Nonetheless, the biological mechanisms and evolutionary processes underpinning such dramatic transformations remain poorly understood; especially in quantitative biomechanical terms^5–8^.

The lineage of Triassic archosaurs leading to sauropods began as quadrupeds, transitioned to bipedalism close to the base of Dinosauria, and then shifted back to quadrupedalism close to Sauropoda^1,2,4,6,9,10^, and in ornithischian dinosaurs independently^11–16^. Apart from these shifts from quadrupedalism to bipedalism and *vice versa* that occurred through the phylogeny of several archosaur lineages, it has been proposed that there were shifts in locomotor stance during the ontogeny of some individual dinosaurian taxa^17–20^.

Ontogenetic changes are crucial to understand heterochronic patterns and the role of the latter in the origin of evolutionary novelties (e.g.^21,22^), such as the evolution of several dinosaur groups^23–25^, including Sauropodomorpha^26–29^. For example, changes in the cross-sectional geometry of femora may be indicative of a shift from bipedal to quadrupedal stance within a few months of hatching, as suggested for *Dryosaurus*^17^; or just the opposite, with femora and tibiae becoming relatively weaker at resisting bending loads with increasing size, concurrent with relative strengthening of humeri, indicating a shift from bipedalism to quadrupedalism as in the hadrosaurids *Maiasaura* and *Iguanodon*^18,19^. The ornithischian *Psittacosaurus* has been hypothesized to have shifted from a quadrupedal stance in hatchlings to bipedalism in adulthood based on differences in limb allometry^20^. In a similar way, it has been suggested, based on changes in forelimb/hindlimb lengths, that the early sauropodomorph *Massospondylus* was quadrupedal in early ontogenetic stages and adopted bipedal stances in adulthood. The ontogenetic shift from quadrupedalism, typical of sauropods, to the facultative or obligate bipedalism of *Massospondylus* (the ancestral condition) led to inferences that quadrupedalism in sauropods could have evolved through heterochrony (i.e., paedomorphosis), by the retention of early ontogenetic features in adults^28,30^ (however, see^31^).

Nonetheless, it has not been tested whether the pattern estimated for *Massospondylus* is applicable to other early sauropodomorphs (largely because of the lack of ontogenetic series) or if it was an ontogenetic trajectory exclusive to its own lineage (or a restricted group to which it belonged, such as massospondylids). Evaluating the generality of shifts in locomotor stance among early sauropodomorphs within phylogenetic context is crucial to test the hypothesis of a paedogenetic origin of quadrupedalism in Sauropoda. Moreover, evidence supporting previous shifting of stance in dinosaurs during ontogeny is based on limb proportions and allometric scaling^28^, but still there is a lack of unambiguous, more direct osteological or biomechanical evidence supporting quadrupedalism at any age in sauropodomorphs, as noted for other dinosaurian groups^14^.

Body mass (BM) and centre of mass (CoM) position are useful when analysed in a phylogenetic context, as previously demonstrated for sauropodomorph dinosaurs^4^. First, there seems to have been a caudad shift of whole-body CoM since the Middle Triassic in Dinosauromorpha, associated with the evolution of bipedalism in various dinosauromorph lineages (or ancestrally). Second, that CoM shift was reversed in Late Triassic sauropodomorphs, matching the evolution of quadrupedalism. Finally, there was a more striking craniad shift in Late Jurassic–Cretaceous titanosauriforms, which included the largest sauropods, with elongate forelimbs and necks^4^. To date, however, quantitative CoM analysis has not been applied to ontogenetic series in sauropodomorph dinosaurs, although qualitative or intuitive inferences have been implied or stated (e.g.^28^). Moreover, to obtain a more complete reconstruction of ontogenetic patterns underlying shifts of locomotor stance in Sauropodomorpha, it is necessary to make comparisons between shifts of locomotor stance along ontogenetic and phylogenetic trajectories, but to evaluate the impact of body segments on CoM (i.e. their first mass moments; e.g.^4,32^) at each ontogenetic stage, which have not hitherto been done.

**Figure 1:**
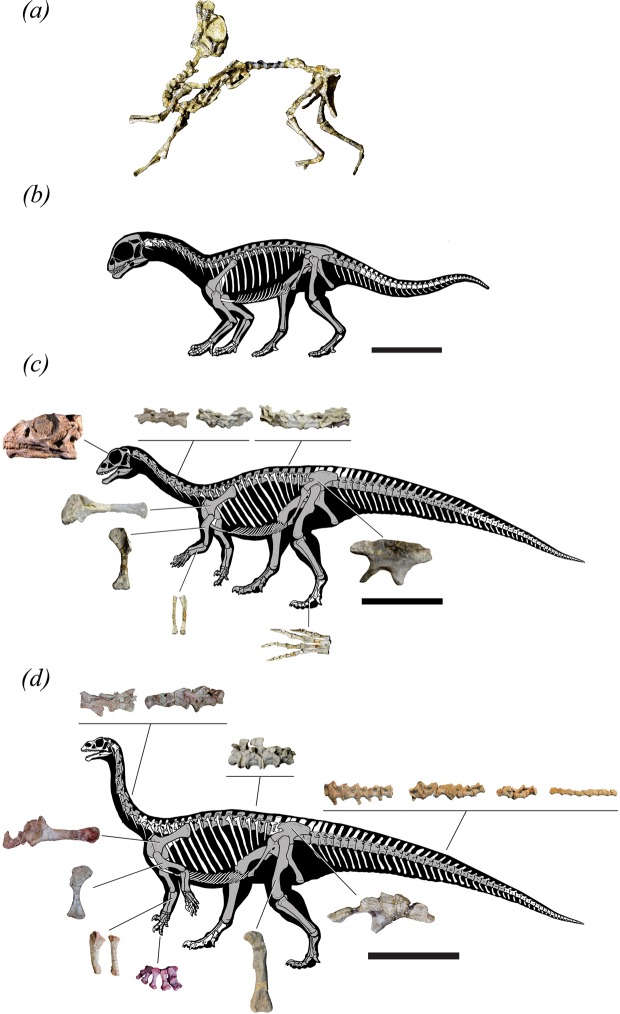
*Mussaurus* specimens. (**a**,**b**) hatchling, (**c**) yearling, (**d**) adult. Scale bars represent 5 cm (**a**), (**b**) 15 cm (**c**) and 100 cm (**d**). To better show isolated bones in (**c**), we used specimen PVL 4587, of the same ontogenetic age as MPM 1813 (except for the ilium, which belongs to MPM 1813). Preserved bones are shaded in grey.

*Mussaurus patagonicus* is an Early Jurassic sauropodomorph from Patagonia (Argentina) known from ontogenetic series ranging from whole skeletons of hatchlings^26^ and one-year old juveniles (“yearlings”)^33,34^ to adult individuals^35,36^ (Fig. 1). The availability of these almost complete skeletons, representing key ontogenetic stages, along with the phylogenetic position of *Mussaurus* (closer to Sauropoda than *Massospondylus*), provide a critical opportunity to illuminate biomechanical and “evo-devo” mechanisms underlying the origin of sauropods^8^.

Here we use full-body digital reconstruction methods to estimate BM and CoM for ontogenetic stages of *Mussaurus* in order to quantify its approximate ontogenetic trajectories and their potential relationship with locomotor stance. We predict that *Mussaurus*’s CoM shifted caudally during ontogeny as the relative sizes of the head/neck and pectoral appendages were reduced whereas the hindlimbs were enlarged. Additionally, we analyse the impact of body segments on CoM across *Mussaurus*’s ontogeny and its consistency with shifts of locomotor stance across sauropodomorph phylogeny.

## Methods

### Bone geometry acquisition

Fossils of the early sauropodomorph *Mussaurus patagonicus* used here comprise specimens of different ontogenetic stages, from post-hatchlings to adults^26,33,35^. Our study focused on the best-preserved and most complete individuals of three ontogenetic stages: a hatchling (MACN-PV 4111), one-year-old juvenile (“yearling”) (MPM 1813) and adult, the latter represented by a composite skeleton of three individuals (MLP 68-II-27-1; MLP 60-III-20-22; MLP 61-III-20-23). X-ray micro-computed tomography scans (Nikon XT 225 ST microCT scanner; University of Cambridge Museum of Zoology) were taken of the entire hatchling and yearlings (Supplementary Table S1).

The hatchling corresponds to one of the seven individuals found together in a nest and described by^26^; the yearling was a composite of two individuals belonging to a block of about 12 individuals discovered in close proximity to each other. Where bones were missing from the individuals, we scaled bones from the other ontogenetic stages so that they would articulate with the corresponding bones. Supplementary Table S2 provides measurements of the individual bones. For caudal vertebrae, the adult individual MLP-61-III-27-1 has 30 preserved elements, but not fully preserved haemal arches (chevrons). For the purposes of our model, a scan of the complete tail of *Plateosaurus* (GPIT 1; from^37^) was rescaled to fit the sacrum and approximate dimensions of the 30 preserved elements of *Mussaurus*. As a result, a complete tail of 40 elements with *Mussaurus*’s proportions was used for the adult model. The yearling preserves the proximal 19 caudal vertebrae. Remaining elements were rescaled from the adult individual to fit the proportions of existing elements. All but six of the caudal vertebrae of the hatchling are absent so these bones were rescaled to represent all 40 vertebrae of the tail. Supplementary Table S3 details the dimensions of representative tail segments. Because the adult skull is missing, a skull of an adult *Plateosaurus* (SMNS 13200; from^37^) was used, which is approximately the same size as *Mussaurus*’. Using the CT scan data from the hatchling and yearlings, the individual bones were segmented using Mimics 18.0 software (Materialise Inc., Leuven, Belgium). A three-dimensional portable surface scanner (NextEngine®, Santa Monica, CA, USA) was used to digitize the adult specimens. Finally, photogrammetry was used to make a 3D model of the yearling’s skull (PVL 4587), using a Nikon® D7000 camera with a Nikkon® 18-105 mm lens and 69 photographs, processed in Agisoft 1.4.1.5925 software.

### Three-dimensional modelling and centre of mass

**Figure 2:**
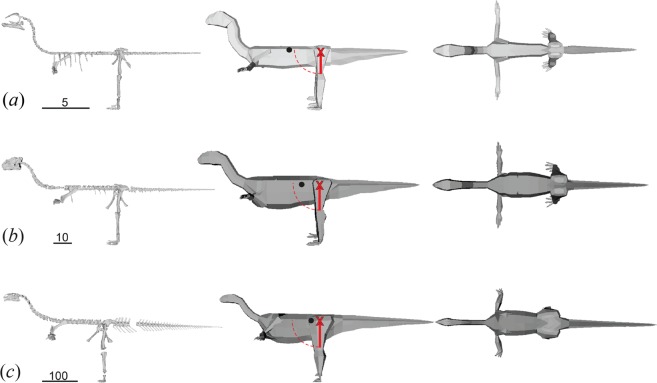
*Mussaurus* skeletons and spline-based models corresponding to: (**a**) hatchling; (**b**) yearling; (**c**) adult. Estimated centres of mass (CoM) are denoted with a black dot relative to femur length from the acetabula (red ‘X’). Bipedal static stability is possible where the CoM is within one femur length (red line) of the acetabula (dashed line), i.e. in the yearling and adult models. Scale bars in cm. Convex hull models are available in Supplementary Information Fig. S1.

Specimens were digitally re-articulated in Blender 2.18 (Blender Foundation, https://www.blender.org/). Where bones from multiple individuals were used, the bones were scaled up to represent the largest individual of that group. The models were then put into a default pose as described by^32^, where the forelimbs were abducted and held horizontally to the ground, the hindlimbs straightened with the digits at 90 degrees to their respective lower legs, and the hindlimbs angled at 15 degrees laterally from the body (i.e. abducted) from the acetabula. The neck was curved into an S-shape such that the final horizontal length was half the original length (Fig. 2), whereas the dorsal and caudal vertebrae were placed into a straightened position with the prezygapophyses and postzygapophyses in articulation.

These posed skeletons were used to fit 3D volumes using spline-based^4,32,38^ and convex hull-based^7,39,40^ digital reconstruction methods to compare results, as detailed below. As the tails of the juvenile and hatchling were derived from only centra (missing the chevrons, neural spines, etc.), the margins of the tail “skeleton” for the spline-based models were delimited from estimates from the *Plateosaurus* tail used in the adult, incorporating the length and angles of chevrons throughout the tail (see Supplementary Table S3). For the convex-hull models, the entire thorax, pelvis, and tail were meshed as a single entity. Thus, the mesh over the tail extended from the distal ischia (ventrally) and pelvis-sacrum (dorsally) to the distal tail tip, encompassing the missing elements.

We then applied two different methods to these models for estimating body mass and CoM. The first of these was a minimum convex hull created using the “convex hull” filter in Meshlab v2016.12 software (Visual Computing Lab, ISTI – CNR, Pisa, Italy)^4,7,40^. Body volume and CoM were output using the “geometric measure” feature for all combined convex hulls. Body mass estimates were calculated by multiplying the volume by densities of either 974.7 kg m^−3^ for the minimum mass or 1181 kg m^−3^ for the maximum mass model (see^4,7^).

The second method was the spline-based method outlined by^32^, which like the convex hull method has been validated on extant species (e.g.^38^ also see Discussion). The spline-based method was furthermore subjected to modifications of body segments allowing for sensitivity analyses representing (as relatively implausible extremes) maximal caudal and cranial masses; maximal dorsal and ventral masses; and maximal and minimal (total body) masses (each involving body segments altered in shape by ±20% from the initial models to bias the masses and CoMs toward extreme values; e.g. larger neck, forelimbs and body, but smaller legs and tail for “maximal cranial” model (see^4,32^). The volumes calculated for each of these models were multiplied by 1060 kg m^−3^ to obtain the masses, but zero-density air cavities in the torso, neck and head were included to represent these empty spaces (following^4,32^). To quantify how each segment influenced whole body CoM, we computed their first mass moments (FMM = segment mass * segment CoM distance from acetabula) using the spline-based models.

Ages were determined by histology in previous studies that showed the juveniles were under one year old^34^, whilst the adult individual was 8+ years old and, owing to the spacing of the lines of arrested growth becoming reduced, was at or reaching adulthood^36^. The smallest individuals reported by^26^ were interpreted as hatchlings given they are larger than the eggs found in close association as well as those found in the fossiliferous locality.

Institutional abbreviations: GPIT – Institute for Geosciences, Eberhard-Karls-Universität Tübingen, Tübingen, Germany (formerly Geologisch-Paläontologisches Institut Tübingen), MLP – Museo de La Plata, La Plata, Argentina; MPM – Museo Regional Provincial “Padre M. J. Molina”, Río Gallegos, Santa Cruz, Argentina; PVL – Instituto “Miguel Lillo”, San Miguel de Tucumán, Tucumán Argentina; SMNS – Staatliches Museum für Naturkunde Stuttgart, Stuttgart, Germany.

## Results

### Body mass and centre of mass

We calculated body mass using two modelling methods: convex-hulling^7,39,40^ and spline-based^4,32,38^ (see Methods). Overall, these methods produced similar results for both BM and CoM changes through ontogeny (Figs 2, S1; Tables 1, 2). The hatchling was around 0.07 kg, with BM increasing by about 100 times in the first year of life, and by >150 times from yearling to adulthood (Table 1). The convex hull models gave varied results relative to the average spline-based models: the hatchling had a 9% greater BM, whereas the yearling and adult respectively had a 1% and 12% smaller BM (Table 1).

**Table 1:** Approximate ontogenetic changes in two parameters of three growth stages of *Mussaurus*: body mass and whole-body CoM position (craniad to acetabula), as estimated by the spline-based reconstruction method (“Maximal mass” and “Minimal mass” models from enlarged/reduced segments, the average of these, and the difference of the average from the Maximal mass model) and by the convex hull method.

	Age (years)	Spline-based reconstruction				Convex Hull
		Maximal	Minimal	Average	Difference	
**Body mass** (**kg**)						
Hatchling	0	0.0908	0.0449	0.0679	0.0229	0.0740
Yearling	1	11.5	5.10	8.30	3.20	8.19
Adult	8+	1937	898.2	1418	519	1257
**CoM** (**% distance from acetabulum to glenoid**)						
Hatchling	0	0.471	0.436	0.453	0.018	0.447
Yearling	1	0.410	0.462	0.436	−0.026	0.308
Adult	8+	0.235	0.242	0.239	−0.0040	0.0990

**Table 2:** Centre of mass (CoM) of the different ontogenetic stages of *Mussaurus* as estimated by both convex hull and spline-based methods.

	Age (years)	Convex hull CoM	Cranial CoM	Caudal CoM	Spline-based average CoM
Hatchling	0	**0**.**447**	0.632	0.245	**0**.**439**
Yearling	1	**0**.**308**	0.522	0.271	**0**.**397**
Adult	8+	**0**.**0990**	0.379	0.0513	**0**.**215**

The CoM values are a proportion of acetabular-glenoid distance, with values closer to 1 being more cranial. Cranial and Caudal CoMs are the two extreme models (“maximal cranial” and “maximal caudal”) from the spline-based methods, and Spline-based average CoM is the mean of the two.

The spline-based models’ CoM of *Mussaurus* shifted from a relatively cranial position in the hatchlings (Fig. 2; Tables 1, 2) to a slightly more caudal position in the yearlings (3–11% closer to the acetabula). By the time *Mussaurus* reached adulthood, the CoM moved even further caudally (> ~20% reduced distance from the acetabula relative to glenoids). The convex hull models’ CoMs were more caudally positioned than in the spline-based models of the yearling and adult, but highly comparable in the spline-based models of the hatchling (Table 2). However, sensitivity analyses showed that all of these estimates overlapped (e.g. a CoM from a more caudally-biased spline-based model had a CoM further caudad than the convex hull’s CoM), as did the maximal and minimal mass models’ CoMs (Table 1).

To stand or move bipedally, the CoM should not be further craniad to the acetabula than the femur length, or else static stability becomes impossible. With a 0.726 m long femur, the CoM of the adult was, depending on methods, 0.12–0.85 femur lengths craniad to the hips. Similarly, in femur lengths the yearling’s CoM was 0.8–1.5 (based on a 0.115 m femur) and the hatchling’s CoM was 0.68–1.8 (based on a 0.0255 m femur) craniad to the hips. Consequently, for the hatchling and yearling there were models (including the average models) for which the CoMs exceeded the femoral length from the acetabula, but in the adult *Mussaurus* model all CoMs were within one femoral length of the acetabula.

**Figure 3:**
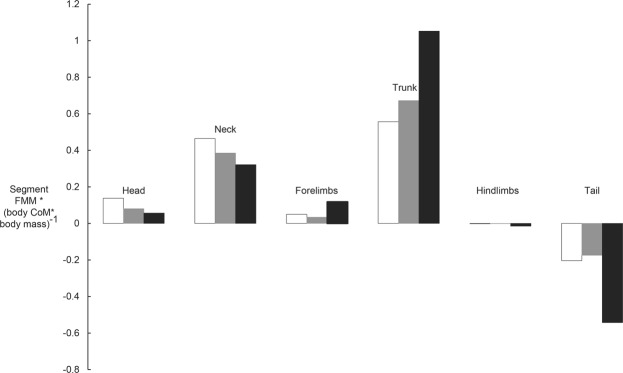
Differences in segmental first mass moments (FMM, calculated by multiplying segment mass by segment CoM distance from origin, located between the acetabula; normalized by body CoM times BM) in the three growth stages of *Mussaurus*, as estimated using the “average” spline-based models (Fig. 2). White, hatchling; grey, yearling; black, adult.

Segmental first mass moments (FMM) generally showed consistent patterns: the trunk and neck (in particular), and head and forelimbs (much less so), shifted the whole-body CoM in a craniad direction the most, whereas the hindlimbs had negligible effects and the tail shifted the CoM in a caudad direction. However, some body segments showed divergent patterns across ontogeny for how their FMM influenced CoM. The head and neck had stronger relative influences for craniad shifts in, proceeding in descending order, the hatchling vs. yearling vs. adult models. In contrast, the more robust trunk, as well as (to a lesser degree) the forelimbs (both exterting a craniad CoM influence), offset by the more massive tail (a caudad CoM influence; supplemented slightly by the hindlimbs), contributed proportionately more to CoM position in the adult *Mussaurus* model (Fig. 3). Hence across *Mussaurus* ontogeny, the results indicated that a smaller head and neck and a larger tail mainly caused the caudad CoM shift estimated here.

## Discussion

**Figure 4:**
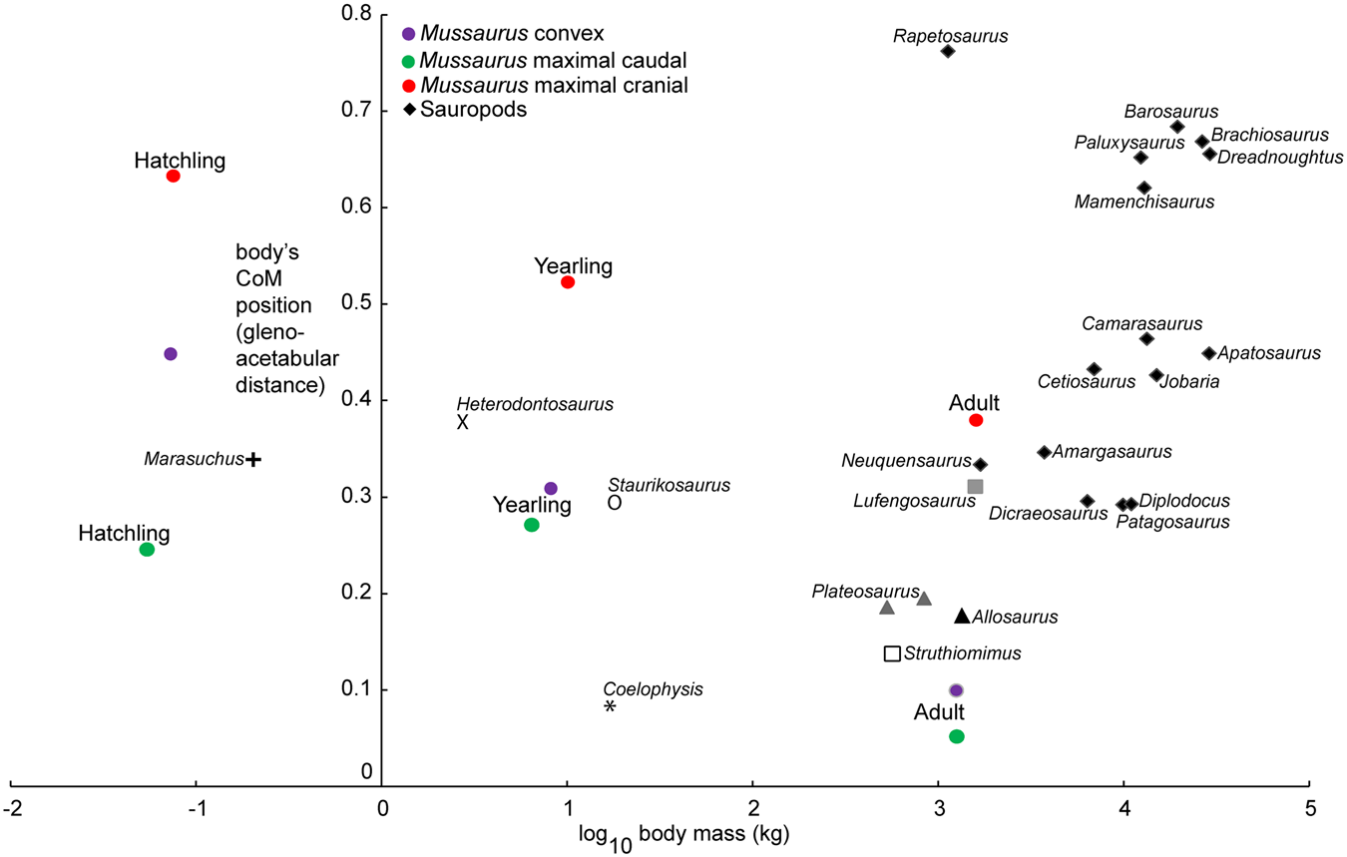
Comparative analysis of whole-body CoM position vs. body mass in Sauropodomorpha, with *Mussaurus* ontogenetic trajectory data (Fig. 3; Table 2) mapped onto interspecific convex hull-based modelling data from^4^ (see Supplementary Table S3). All sauropods included clearly were quadrupedal, whereas *Plateosaurus* and possibly *Lufengosaurus* are considered bipedal as adults (and have CoM values close to that of the *Mussaurus* adult). Three theropods (unambiguous bipeds) of varying masses (spline-based model data from^4,32^) are also included for comparisons. *Mussaurus* spline-based model data points are shown for the maximal cranial and maximal caudal models (Table 2), which refer to the modifications of body segments in sensitivity analyses representing (as relatively implausible extremes) maximum caudal and cranial CoMs.

Our computational models of ontogeny revealed novel information regarding the life history of *Mussaurus patagonicus* and its shift in locomotor stance during ontogeny. Our *a priori* prediction that reduced pectoral appendages and head/neck vs. enlarged pelvic limbs drove the CoM caudally across the ontogeny of *Mussaurus* was only partly supported. The main segmental influences on CoM, as Fig. 3’s FMM data show, were indeed from a reduced head/neck but more from an enlarged tail than hindlimbs (or reduced pectoral appendages). We inferred that the caudad shift of the CoM from hatchling toward adulthood involved a shift from quadrupedal to bipedal stance for three reasons. First, the CoM of hatchlings was too far forward for the short hindlimbs to place the feet under the CoM in a statically stable bipedal pose (except in the most extreme, more implausible “maximal caudal” model, where the hatchling CoM was <1 femur length craniad to the acetabula; see Table 2). Second, the position of the CoM of *Mussaurus* hatchling (about halfway along the trunk; and more than one femur length craniad to the acetabula) corresponded to the CoM recently estimated for several quadrupedal sauropods, showing a similar position to that of *Camarasaurus*^4^ (Fig. 4) and far from any bipeds, supporting quadrupedal stance in *Mussaurus*’s early ontogenetic stages. Third, prior analyses have shown that adults, at least, were unlikely to be able to use their forelimbs in locomotion^8^ as inferred for other early sauropodomorphs (e.g.^37,41^).

Whilst our study is not a test of the two volumetric methods used, it is useful to discuss the two methods used herein and technical issues that underlay our motivation for using both methods. Both convex hull modelling and spline-based approaches are commonly used for estimating the mass of extant and fossil species, and tend to produce roughly similar body mass estimates; as in this study. Convex hull modelling is quick in comparison to spline-based models and is relatively more objective^42^. However, we have concerns that convex hull models, especially for sauropsid tetrapods with large tails and pelvic limbs, can produce CoM estimates that are inaccurate for those segments and for the whole body (e.g. if hulled too tight to the tail, an estimated CoM that is too far craniad than a reconstruction based on fleshy rather than skeletal tail anatomy). To date, validation studies of extant tetrapods using convex hulls have focused on mammals and birds^40,43,44^ that have relatively small tails, not on modelling thick tails that connect to the pelvic limb. One study of an extinct lion also has cautioned that the mass and CoM of the proximal limb are error-prone in convex hull models^45^ due to the inherent limits of wrapping the bones vs. bulky proximal limb muscles. Prior studies of sauropsid segment dimensions have noted the wide deviation of the actual tail (and pelvic limb) from the actual skeleton^38,46,47^. Some convex hull-based modelling of tails in dinosaurs seems to have implicitly attempted to compensate for this deviation by extending the hulls from the pelvis to the tail, but methods vary (e.g.^39,40^). Here we used a different method that hulls the tail as a single entity extending from the pelvis (bounded by the ischium ventrally, transverse processes laterally, ilium and neural spines dorsally and distally the terminal caudal vertebra), which seems to help our overall CoM patterns overlap (for convex hull vs. spline-based models), by producing tail morphologies that are more anatomically plausible in light of existing data from phylogenetic bracketing. Further validation work should be carried out to test and refine best practices for convex hull modelling in such species.

A vexing problem for assessing quadrupedal stance is that the possibility of forelimb usage in hatchlings, in particular, is difficult to evaluate because of the poor ossification of joint surfaces used to infer mobility in adults^8^. Such poor ossification of joint surfaces is a widespread condition among preserved dinosaur remains of early ontogenetic stages^20,30^. The close association of multiple articulated skeletons of hatchlings along with eggs and eggshells^26^ suggests that these individuals may have been altricial and therefore a quadrupedal stance need not have involved efficient quadrupedal locomotion moving far from the nest. There are two more reasons that the inference of quadrupedal stance in the hatchlings is reasonable. First, the preserved craniomedial position of the radius relative to the ulna in the articulated skeleton PVL 4068 (Fig. 1) is congruent with, at least, a semi-pronation of the forelimb^8,48^. Second, considering the appreciable morphological disparity between *Mussaurus*’s hatchlings and adults, and assuming a rapid rate of morphological change across ontogeny^34^, even if adult *Mussaurus* were incapable of full manus pronation, as previously proposed for some early sauropodomorphs^41^, that does not mandate that hatchlings were similarly incapable of pronation. Hence overall we contend that our conclusion is the most plausible one in light of all available evidence. Because of uncertainty in the models’ estimates, inferences on the locomotor stance in the yearling are ambiguous. Nonetheless, we speculate that the yearlings at least had a facultatively bipedal stance, based on the ~3–11% closer CoM distance to the acetabula, bringing the CoM within 1 femur length of the hips; unlike in the hatchlings (Figs 2, S1).

Although a recent analysis conducted on ornithischian dinosaurs concluded that a caudally positioned CoM does not necessarily imply bipedal locomotion^49^, it is still the case that a CoM too far cranial to the hips will mandate quadrupedal stance. Multiple lines of evidence support a bipedal stance in adult specimens of *Mussaurus*, coincident with its more caudally positioned CoM, including proportionally shorter forelimbs (shifting from forelimb/hindlimb length ratios of 0.76 to 0.55 from hatchlings to adults; Supplementary Table S2), an arched metacarpus, a recurved and medially divergent first digit of the manus, and absence of a fully pronated manus (evidenced by an interlocking radius and ulna)^8,41^. Moreover, the ontogenetic changes described herein for *Mussaurus* complement previous studies in which *Mussaurus*’s adult specimens were inferred as bipeds based on minimum limb bone shaft circumferences^10^.

The pattern estimated across ontogeny in *Mussaurus* reveals that at least some early sauropodomorphs experienced a transition from quadrupedal to bipedal within their life history. Although previous studies based on limb proportions suggested that *Massospondylus* also experienced a locomotor shift during ontogeny^28^, novel information on inner ear morphology does not support such a locomotor shift^31^. Other studies have questioned the ability of inner ear morphology to infer head, neck or body orientation in tetrapods^50,51^, but further analysis of this problem and data on ontogenetic changes of inner ear morphology in *Mussaurus* would be valuable. Consequently, and considering the available evidence to date, it is not possible to elucidate if the locomotor shift estimated for *Mussaurus* corresponded to a trend or an exception among early sauropodomorphs. However, if it was actually a trend it contrasts with the available evidence in sauropods indicating a single stance (quadrupedal) across all known ontogenetic stages^52^. Nonetheless, multiple lines of evidence support an evolutionary change of stance along sauropodomorph phylogeny, from bipedal (in early sauropodomorphs) to quadrupedal (in sauropods/Eusauropoda; Fig. 4), ranging from different studies that concluded adult forelimbs were unable to have acted in a major locomotor role in early sauropodomorphs^8,37,41^ to an analysis that reconstructed a progressively caudal to cranial shift of CoM through sauropodomorph phylogeny^4^. One exception to this pattern is the clade Lessemsauridae (either sauropods or close-to-Sauropoda, depending on the Sauropoda definition), whose members’ body masses are estimated at over 10 tonnes, with putatively obligate quadrupedal stance involving heavily built, moderately flexed forelimbs^9,10^. Biomechanical studies of the latter clade could be particularly informative.

While Fig. 4 shows that all bipedal taxa had a CoM <40% of gleno-acetabular distance craniad to the hips, and no quadrupeds had a CoM <25% of gleno-acetabular distance craniad to the hips, there is an interesting zone of overlap between unambiguous bipeds, quadrupeds, and taxa of ambiguous locomotor stance between these zones. Such ambiguity and overlap of CoM “morphospace” should be more closely examined, especially in the context of limb lengths and other important parameters (e.g. femur length as discussed here), and where feasible across ontogeny and phylogeny.

The ontogenetic shift from quadrupedal to bipedal stance in early sauropodomorphs was previously regarded as providing evidence that the quadrupedal stance in sauropods evolved through paedomorphosis^28^. Our analyses based on 3D skeletal models revealed a more complex evolutionary scenario. Despite the CoM analysis suggesting a quadrupedal to bipedal ontogenetic shift in *Mussaurus* that superficially appears as inverse to the bipedal to quadrupedal shift in the phylogeny of Sauropodomorpha, the skeletons of adult sauropods and juvenile early sauropodomorphs are built in different ways and cannot be equated. In particular, our analysis of FMMs revealed major differences in how the relative dimensions of certain body segments of the body plan of *Mussaurus* developed (Fig. 3). Even though the proportional reduction of the head/neck region in *Mussaurus* adults, and its correlation with a shift of locomotor stance throughout ontogeny, fit those previously proposed for *Massospondylus*^28^, we found that the enlarged tail had the dominant influence on moving CoM caudally in the late ontogeny of *Mussaurus*, constituting a previously unreported key factor (Fig. 3). Furthermore, our study is the first to analyse the effects of all major body segments on CoM (i.e. using FMMs) and thus locomotor stance during the ontogeny of a non-avian dinosaur. Our results somewhat parallel those of^4,32^, who uncovered evidence that a reduced tail in theropods on one hand, and an enlarged head/neck in sauropodomorphs on the other, were correlated with craniad shifts of CoM across macroevolutionary transitions. It is noteworthy that the hatchling’s tail was reconstructed from the juvenile’s tail and scaled proportionally. This resulted in a maximal tail size for this ontogenetic stage (with a FMM similar to the yearling’s; Fig. 3) and, despite this, the CoM was strongly cranially positioned. Any smaller tail size would have resulted in an even more cranial CoM, bolstering our finding that a caudal body CoM was largely driven by increasing tail size (along with decreasing head and neck sizes). The tail’s important role in this transition fits its increasing importance in supporting bipedal locomotion via the tail-based caudofemoralis muscle (e.g.^53^). Thus, although previous studies have emphasized the influence of hindlimb/forelimb length proportions in sauropodomorph stance^20,28^, our study indicates that the relative development of tail (and neck) was more influential in constraining/determining the locomotor stance in this clade, in both ontogenetic and phylogenetic changes.

The ontogenetic patterns of body shape and CoM in *Mussaurus* are somewhat counter to those estimated for the theropod *Tyrannosaurus* (albeit across a narrower ontogenetic spectrum). In the latter taxon, the torso enlarged whereas the limbs reduced relative to body size, concurrent with an approximate craniodorsal shift of the body’s CoM^54^; as in extant archosaurs^38^. This contrast indicates hitherto unappreciated diversity in ontogenetic patterns of body shape and CoM in Archosauria. More such studies of biomechanically-linked parameters across growth series of archosaurs would be valuable to infer how much diversity exists.

## Supplementary information

Supplementary information

## Data Availability

The digital models, including skeletons, of *Mussaurus patagonicus*, are available at https://figshare.com/s/264325d699de7323aa4e.
