# Supplementary material for: Ontogenetic changes in the body plan of the sauropodomorph dinosaur Mussaurus patagonicus reveal shifts of locomotor stance during growth
Source: Sci Rep. 2019 May 20;9:7614. doi: 10.1038/s41598-019-44037-1 (PMC6527699; doi:10.1038/s41598-019-44037-1)
Supplement: Supplementary file 1 — Supplementary information [file 41598_2019_44037_MOESM1_ESM.pdf]

Supplementary Information

Ontogenetic changes in the body plan of the sauropodomorph dinosaur *Mussaurus patagonicus* reveal shifts of locomotor stance during growth

Alejandro Otero[1, 2*], Andrew R. Cuff[3**], Vivian Allen[3], Lauren Sumner-Rooney[3, 4], Diego Pol[2, 5], John R. Hutchinson[3]

[1]División Paleontología de Vertebrados, Museo de La Plata, Paseo del Bosque s/n, La Plata (1900), Argentina.

[2]CONICET - Consejo Nacional de Investigaciones Científicas y Técnicas, Argentina.

[3]Structure and Motion Laboratory, Department of Comparative Biomedical Sciences, The Royal Veterinary College, Hatfield, Hertfordshire, United Kingdom.

[4]Oxford University Museum of Natural History, Oxford, United Kingdom.

[5]Museo Paleontológico Egidio Feruglio, Trelew, Argentina.

*alexandros.otero@gmail.com

**acuff@rvc.ac.uk (dual corresponding authors)

Supplementary Tables

Supplementary Table S1. X-ray micro-computed tomography settings used for *Mussaurus*'s specimens.

Specimen	Voltage (kV)	Amperage (μA)	Voxel size (mm)	
MACN–PV 4111	150	800	0.101	Hatchling
MPM 1813	150	815	0.109	Pelvis/hindlimbs yearling (2 scans, 2 individuals)
MPM 1813	190	1020	0.125	Scapula/spine/forelimb

Supplementary Table S2. Measurements of *Mussaurus* bones. FL, forelimb (humerus plus radius); HL, hindlimb (femur plus tibia). Measurements are in mm.

Ontogenetic stage	Specimen #	Skull	Scapula	Humerus	Radius	Ulna	Femur	Tibia	Fibula	FL	HL	FL/HL ratio
Hatchling	PVL 4068	37	30	27	17	18	30	28	28	44	58	0.76
Hatchling	MACN 4111	31.2		23.5	14.8	15.2	27.0	27.6		38.3	54.6	0.70
Yearling	MPM 1813	95	103	95	65	65	112	105	100	160	217	0.74
Yearling	PVL 4587	97	112	113	75	78		138	137	188		
Adult	MLP 68-II-27-1	-	600	460	240	278	770	510	525	700	1280	0.55
Adult	MLP 60-III-20-22	-	-	-	-	-	800	579*	543	-	-	-

*incomplete

Supplementary Table S3. Measurements of *Mussaurus* reconstructed tail skeletons. Caudal vertebrae dimensions, and total proximodistal tail length. Columns for each ontogenetic stage show measurements in mm (on left), and then ratios of those measurements to tail length (on right). "Chevron estimate" is the dorsoventral depth of the chevron (haemal arch) reconstructed from *Plateosaurus* data (see Methods).

	Hatchling		Yearling		Adult	
**Caudal 1**						
craniocaudal	3.22	0.036	14.0	0.032	88.3	0.028
mediolateral	3.41	0.038	11.7	0.027	110	0.035
dorsoventral	2.70	0.030	17.3	0.040	114	0.036
**Caudal 20**						
craniocaudal	1.74	0.019	8.78	0.020	69.5	0.022
mediolateral	1.77	0.020	5.70	0.013	47.4	0.015
dorsoventral	1.39	0.016	6.03	0.014	40.5	0.013
chevron estimate	5.27	0.059	20.2	0.046	117	0.037
**Caudal 40**						
craniocaudal	0.55	0.0062	4.48	0.010	39.4	0.013
mediolateral	0.39	0.0043	1.99	0.0046	11.4	0.0037
dorsoventral	0.43	0.0048	2.34	0.0054	11.2	0.0036
**Tail length**	89.3		436		3130	

Supplementary Table S4. Body mass and CoM values (in % glenoacetabular distance as per Tables 1,2) for various saurischian dinosaurs; as used in Figure 4. All data are convex hull models from [4], except *Allosaurus* and *Struthiomimus* spline-based models from [32], in addition to *Mussaurus* spline-based maximal caudal and maximal cranial CoM models.

**Taxon**	**Mean body mass (kg)**	**Log_{10} body mass**	**Craniocaudal CoM (%)**	**Locomotor category**
Mussaurus hatchling	0.0553	-1.257	0.25	Quadruped
Mussaurus yearling	6.51	0.8132	0.27	Biped?
Mussaurus adult	1263	3.101	0.051	Biped
Mussaurus hatchling	0.0765	-1.116	0.63	Quadruped
Mussaurus yearling	10.2	1.007	0.52	Biped?
Mussaurus adult	1608	3.206	0.38	Biped
Mussaurus hatchling	0.0737	-1.132	0.45	Quadruped
Mussaurus yearling	8.19	0.9133	0.31	Biped?
Mussaurus adult	1257	3.0993	0.10	Biped
Marasuchus	0.206	-0.6853	0.34	Biped
Heterodontosaurus	2.76	0.4414	0.38	Biped
Staurikosaurus	17.4	1.2415	0.29	Biped
Coelophysis	16.9	1.2281	0.084	Biped
Allosaurus	1335	3.1256	0.18	Biped
Struthiomimus	567.8	2.7542	0.14	Biped
Plateosaurus	844	2.9	0.19	Biped
Lufengosaurus	1593	3.2	0.31	Biped?
Cetiosaurus	6930	3.8	0.43	Quadruped
Patagosaurus	9920	3.9	0.29	Quadruped
Mamenchisaurus	12900	4.1	0.62	Quadruped
Jobaria	15100	4.1	0.43	Quadruped
Apatosaurus	28900	4.4	0.45	Quadruped

Barosaurus	19500	4.2	0.68	Quadruped
Diplodocus	11000	4.0	0.29	Quadruped
Amargasaurus	3740	3.5	0.35	Quadruped
Dicraeosaurus	6380	3.8	0.30	Quadruped
Camarasaurus	13300	4.1	0.46	Quadruped
Brachiosaurus	26500	4.4	0.67	Quadruped
Paluxysaurus	12400	4.0	0.65	Quadruped
Dreadnoughtus	29100	4.4	0.65	Quadruped
Rapetosaurus	1130	3.0	0.76	Quadruped
Neuquensaurus	1690	3.2	0.33	Quadruped

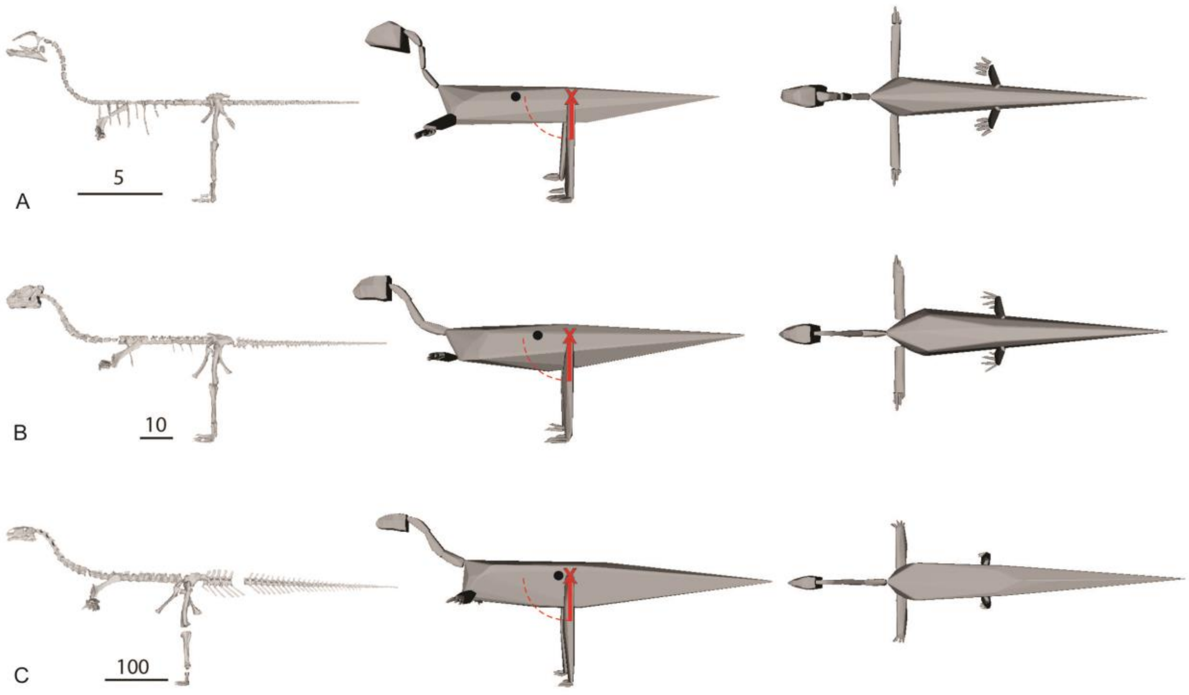

Figure S1. *Mussaurus* skeletons and convex hull models corresponding to: A, hatchling; B, yearling; C, adult. Estimated centres of mass (CoM) are denoted with a black dot relative to femur length (red line) from the acetabula (red 'X'). Bipedal static stability is possible where the CoM is within one femur length of the acetabula (dashed line), i.e. in the yearling and adult models. Scale bars in cm.
